# Patient-centredness: meaning and propriety in the Botswana, African and non-Western contexts

**DOI:** 10.4102/phcfm.v6i1.554

**Published:** 2014-02-20

**Authors:** Vincent Setlhare, Ian Couper, Anne Wright

**Affiliations:** 1 Department of Family Medicine, University of Botswana, Botswana; 2 Department of Family Medicine, University of the Witwatersrand, South Africa

## Introduction

Patient-centredness is a key principle in Family Medicine. It is covered in a Family Medicine textbook by McWhinney^1^ and textbooks edited by Goh et al.,^2^ Rakel and Rakel^3^ and Mash and Blitz-Lindeque,^4^ to name but a few. Patient-centredness (PC) is an extension of the biopsychosocial approach to patient care which was championed by Engel.^5^

The term ‘patient-centredness’ was coined by Balint to emphasise that patients should be treated as unique individuals and was used initially to describe how physicians should interact and communicate with patients.^6^ From highlighting and emphasising the patient’s agenda and appropriate communication skills in doctor–patient interactions, PC grew to include optimal patient–healthcare system interactions. This established the meaning of PC which was distilled by McWhinney as ‘seeing the illness through the patient’s eyes’.^7^

A method of practising PC was then described as paying attention to ‘patients’ cues and behaviour’ and also referred to need for the physician to provide an environment that is conducive to patients’ full and free expression.^8^ Others outlined the method as ‘exploring the illness experience, understanding the whole person, finding common ground regarding management, incorporating prevention and health promotion, enhancing the doctor–patient relationship, and being realistic about the doctor’s personal limitations’.^9^ Variations of this method are described and they have a similar outline.^10,11,12^

The Institute of Medicine, an American non-profit, non-governmental organisation, advises that patient-centredness should be ‘responsive to and respectful of the individual patient’s preferences, needs and values while ensuring that the patient’s values guide clinical decisions’.^13^ The doctor’s awareness of his personal influence and subjectivity promotes patient-centredness.^14^ The doctor or healthcare worker should be aware of their considerable power to influence patients and try to minimise its use. They should also guard against their biases and any vested interests that may constrain their patients’ preferences.

This is the Eurocentric model of PC which is now taught in Botswana and other non-Western settings. The Eurocentric meaning and operationalisation of PC, though based on research in Western contexts, seems to be accepted universally. The universal appropriateness of the meaning and application of the Eurocentric model of PC needs to be backed by evidence from research in non-Western contexts. This article attempts to show how the understanding and practice of PC may be different in other regions. It also hopes to stimulate debate and research on PC in non-Western contexts.

Patients bring expectations to a consultation.^15^ Fulfilment of these expectations is an indication that patient-centredness is likely to have been applied, thus patient satisfaction may be used as a measure of PC.^16^ This tool can be used across settings to explore the qualities, attributes, processes, actions and features of a healthcare system or healthcare worker that are deemed satisfactory by patients. This research may yield health worker–patient interaction models that are context specific and setting appropriate.

## Context is important

Contextual differences between regions suggest that the global appropriateness of the Eurocentric model of patient-centredness is questionable.^9,14,17^ A patient’s culture shapes how they see themselves and environments shape who we are, what we expect and what is expected of us.^18^ Patient-centredness may mean different things in different settings. It may be applied differently in various contexts. The term PC does not exist in Setswana and some other languages, but an interpretation of the sense of the term may be possible. The absence of this term may mean the absence of this construct in non-Western regions.

**FIGURE 1:**
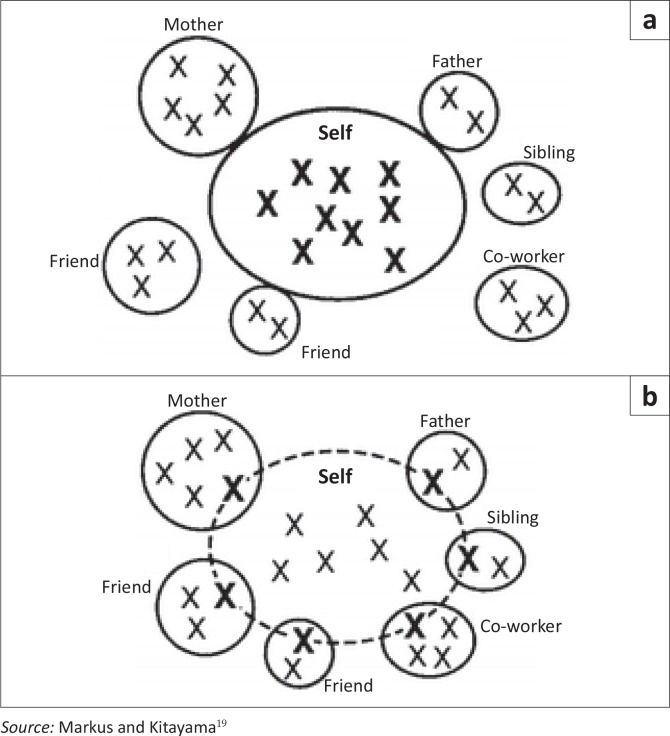
Construals of Self, (a) Independent view of self, (b) Interdependent view of self.

Who we are is also a product of who we understand ourselves to be – the *construal of self* (see Figure 1). The *dominant construal of self* differs between Western and other contexts.^19^ The Western individual sees himself or herself as being an independent agent. They see themselves as complete individuals and promote their personal interests unashamedly, placing emphasis on out-competing others.

In most non-Western societies the ‘individual’ is more integrated with the significant others with whom they have an interdependent relationship. This interdependent self incorporates significant others and contexts into their persona. The interdependent person is inclined to do what is best and appropriate for significant others in a given context, aiming for a harmonious environment. They are likely to align their interests with those of their significant others. These different construals of self have cognitive, emotional and motivational consequences such that phenomena may be seen differently by, evoke different emotions in and elicit varying responses from patients in different regions and contexts. For example, a young person who avoids prolonged eye contact may be perceived to be devious in Western contexts and yet be seen as being polite and well bred in the African context. Similarly, in an African context, when a grown man cries he is seen as being weak and bringing shame to his family and may be given a slap or a scolding for such unmanly behaviour; in a Western context, he may be seen as sensitive and his family may feel proud of him.

Cultural sensitivity in patient-centredness seems to concern itself with the superficial manifestations of culture and not its underlying determinants. Addressing people in a certain way, shaking their hand or not shaking their hand, dress codes and dances all have their place, but are merely window dressing. Markus and Kitayama’s work^19^ points us toward the deeper-seated reasons that explain what we do.

## The African context

Africans in general see themselves as members of tribes and clans rather than as independent agents. An African largely finds identity and value through his community (*motho ke motho ka batho*/*umuntu ngu muntu nga bantu*) and subsumes or aligns individual interests and satisfaction with community preferences –the *interdependent self-construal* approach. The patient’s wishes or agenda as communicated to the doctor sometimes reflect the wishes and agenda of their relatives or clan. Talking to a patient is, in reality, talking to the consensus view of many significant others. Procrastination of the most urgent decisions, as well as inexplicable choices made by patients, may be because the patient is either subconsciously or consciously trying to align disparate interests. Indecisiveness may be because of consultative processes playing themselves out within the family or clan.

As a rule, African communities are highly hierarchical. Elders are to be obeyed and respected. In these communities, life must comply with the norms of the past.^20^ An example of this is where a married woman may prefer not to practise safe sex because her kin want offspring.

Most Africans are born into traditional health systems that interweave health and healing with cosmology and religion.^21,22^ To the African, medicine or healing goes beyond mere healing of diseases and illnesses; it focuses on harmonising relationships amongst the living and between the living and the dead. It permeates the whole fabric of society as it is involved in agriculture, animal husbandry, witchcraft, protection against evil and infliction of evil. It is expressed in taboos, ethics and acceptable social behaviour.

Traditional health practitioners (THPs) are the custodians of this traditional health system and culture. They are in touch with the gods, ancestors and spirits^23^ and are usually called to this profession by these entities. The gods or spirits help them to diagnose and treat diseases.^23^ Patients in this context find it difficult to ‘self-diagnose’ or exchange thoughts with the THPs on what they think could be wrong with them, let alone discuss the treatment. They feel that this would imply that they feel that they have been empowered by the gods to diagnose and treat themselves and that consulting a THP would not be necessary. Many people still go to THPs when they are ill.^24,25^ Traditional medicine is a powerful institution amongst Africans, including Batswana. It has existed for a long time and involves therapeutics, religion and politics.^26^

## The Botswana context

The context that Batswana live in shapes their behaviour, beliefs and expectations. It influences what they expect of doctors and how they behave in the consultation scenario. They carry their beliefs with them and these have an impact on their interaction with modern Western medicine and doctors.

Batswana have lived a traditional, rural life for ages but since gaining independence, Botswana has changed a lot. Most Batswana remain embedded in their culture. They do not consider the towns and cities as their homes but as places where they stay for employment. During public holidays and weekends, they go home to their homes (the rural areas) to enjoy the company of their extended families. When they die, many prefer that their bodies be taken to their rural homes for burial. Most Batswana still believe in *badimo* (ancestral spirits) and *Modimo* (the Creator).^27^ Many Batswana believe in witchcraft, casting of spells, traditional medicine and the power of supernatural agents intervening in their lives.^28^ They respect and hold dear the wisdom of grandparents, parents, elders and the customs and practices that have been passed from generation to generation. Respect for elders can be ascribed to the fact that elders are sometimes acknowledged as *badimo* (dead relatives = ancestral spirits) even though they are still alive,^27^ because they are thought to have easier access to their ancestral spirits. The advice of traditional doctors or traditional midwives is thus taken seriously because of their connection with *badimo*. Educated Batswana and Christians do not seem to escape this bind^29^ – they cannot escape because their culture and its beliefs are part of who they are.^20^ Extricating oneself from this cultural system makes people feel vulnerable.^20^ This describes what Horton calls a closed society, in which one does as others do or risks being a social misfit.^20^

This is not to deny that the culture of Africans and Batswana is evolving. It is to emphasise that it is not yet a culture whose norm is to question the *status quo* and embrace change (open society). It is, rather, a culture that questions departure from the *status quo* and is enamoured with the past.^20^ It is also useful to note that city dwellers and so-called sophisticated Africans revert to traditional health practices when confronted with confounding situations.^23^

Africans and Batswana have thus been shaped by a different culture and history. They see themselves and the world from this different viewpoint. They may have different perceptions, emotions and actions that could be unfamiliar to outsiders.

## An Afrocentric model and its pertinent issues

This is the baggage that an African patient brings to the consultation room – her/his interdependent self-construal, her/his his traditions, belief systems, institutions and history. The African patient’s reluctance to engage meaningfully with regard to diagnoses, treatments and envisaged outcomes frustrates the patient-centred model as currently described. This reluctance may be because of respect for the doctor, whose profession and prominence is revered. The interdependent persona may fail to take a doctor’s enquiries at face value. In seeking to be harmonious, appropriate and sensitive to the doctor’s and significant others’ feelings, answers may be given that seem stupid and do not serve the patient’s best interest. Enquiry about expectations is often viewed by patients as being rhetorical. Ensuring that the patients’ values and preferences guide clinical decisions may be problematic because of paradigm incongruence between healthcare workers (HCWs) and patients. HCWs may misunderstand such values and preferences because of their immersion in or conversion to different values and beliefs. Building meaningful adult relationships between HCWs and patients in societies where patients are family- and clan-accountable can be a challenge. An HCW–patient interaction model suitable for Botswana, Africa and the rest of the non-Western world should be birthed in the daily interactions of doctors and/or HCWs and patients in these contexts. Such a model has to address the impact of political, economic, educational, racial, and tribal factors, both past and present, on HCW–patient interaction and healthcare. It has to harness cultural realities and dominant self-construal paradigms so that they promote health delivery. This model has to be serious about the engagement and incorporation of traditional health practice and its practitioners, as is happening in Glasgow,^30^ Singapore^31^ and China.^32^

Societies within different regions and countries are not uniform. The self-construal of rural people in the Western world may be *interdependent* whilst that of urban elites in other regions may be *independent*. The role of religion, tribe and class in doctor–patient interactions is of great relevance.

There are challenges to birthing this Afrocentric model of HCW–patient interaction. Africa is populated by people of Bantu, Negroid, Arabic, European and other stock. These people live in countries with different tribes, languages, histories, levels of development and institutions. There are significant differences within countries as manifested by civil wars and economic imbalances, so a model that would be suitable for the whole of Africa may not exist. Development of community-, country-, or regionally-appropriate HCW–patient interaction models may be more realistic. All these issues need to be addressed by research aimed at finding HCW–patient interaction models or paradigms that are context appropriate.

## The challenge

The challenge is for clinicians and scholars in Africa and the non-Western world to research doctor- or HCW–patient interaction in their specific contexts. It may help for these researchers to be aware of and bracket the influence of the Eurocentric model of doctor–patient interaction (the patient-centred approach). It may be wise not to do a literature review before commencing on this research^33^ as this could impose Eurocentric-biased findings on the research data generated from observations in a non-Western context. It is hoped that such research will yield doctor- or HCW–patient interaction models that are context appropriate for the various African, Asian, South American and other communities. This research may yield different meanings, interpretations and understandings of patient-centredness. It may also yield a different HCW-patient interaction model. The worth of this research and the conclusions thereof will be in the extent to which patient care is improved, as judged by the patients themselves.

## Conclusion

Patient-centredness is a Eurocentric social construct which is appropriate for the context in which it was birthed through research. Patients in Botswana do not, however, seem to fit into this model. Meaningful discussion with these patients about a possible diagnosis or line of treatment is often difficult and this may be due to regional differences in *construals of self*, culture and contexts. Cultural tinkering of patient-centredness (as presently understood) does not seem to address the difficulties that arise in applying aspects of PC.

People in different regions see themselves and the world differently. They understand, feel and act in a way that is congruent with how they fit into their lived contexts. Patient-centeredness may thus be understood and applied in a manner that makes sense in one region, but which may not be appropriate in other regions.

These concerns and observations highlight the need to explore the meaning and application of PC in non-Western settings. Through research, healthcare workers (doctors, nurses and community health workers) in different communities should work to find context-appropriate and evidence-based models of healthcare worker–patient interaction (PC or other) that enhance both healthcare delivery and patient satisfaction.

Researchers may wish to know how to determine the dominant construal of self in a region. They may also wish to know the nature of HCW–patient interaction that serves the best interest of patients of different dominant construals of self in their contexts. They may want to know the factors that affect patient satisfaction. It is hoped that such research may open more avenues of research that may help to build a *corpus* of evidence that will result in better healthcare and more effective HCW–patient interaction models in non-Western settings.

## References

[1] McWhinney IR A textbook of family medicine. 2nd ed New York, Oxford: Oxford University Press; 1997.

[2] Goh L, Azwar A, Wonodirekso S, editors A primer on family medicine practice. Singapore: Singapore International Foundation; 2004.

[3] Rakel RE, Rakel DP, editors Textbook of family medicine. 8th ed Philadelphia: Elsevier Saunders; 2011

[4] Mash B, Blitz-Lindeque J, editors South African family practice manual. 2nd ed Pretoria: Van Schaik Publishers; 2006.

[5] Engel GL From biomedical to biopsychosocial. Being scientific in the human domain. Psychosomatics. 1997;38(6):521–528. http://dx.doi.org/10.1016/S0033-3182(97)71396-3 9427848 10.1016/S0033-3182(97)71396-3

[6] Saha S, Beach MC, Cooper LA. Patient centeredness, cultural competence and healthcare quality. J Natl Med Assoc. 2008;100(11):1275–1285. PMid: PMCid:19024223 10.1016/s0027-9684(15)31505-4 PMC2824588

[7] McWhinney I The need for a transformed clinical method In: Stewart M, Roter D, editors Communicating with medical patients. London: Sage Publications, 1989; p. 25–42.

[8] Levenstein JH, McCracken EC, McWhinney IR, et al. The patient-centred clinical method. 1. A model for the doctor-patient interaction in family medicine. Fam Pract. 1986;3(1):24–30. http://dx.doi.org/10.1093/fampra/3.1.24, PMid:3956899 10.1093/fampra/3.1.24

[9] Stewart M, Brown JB, Weston WW, et al. Patient-centered medicine: transforming the clinical method. London: Sage; 1995.

[10] Mead N, Bower P Patient-centredness: a conceptual framework and review of the empirical literature. Soc Sci Med. 2000;51(7):1087–1110. http://dx.doi.org/10.1016/S0277-9536(00)00098-8 11005395 10.1016/s0277-9536(00)00098-8

[11] Stewart M, Brown JB, Donner A, et al. The impact of patient-centered care on outcomes. J Fam Pract. 2000;49(9): 796–804. PMid:11032203

[12] Kleinman A, Eisenberg L, Good B. Culture, illness, and care: clinical lessons from anthropologic and cross-cultural research. Ann Intern Med. 1978;88(2):251–258. http://dx.doi.org/10.7326/0003-4819-88-2-251, PMid:626456 10.7326/0003-4819-88-2-251

[13] Naik A. On the Road to Patient Centeredness. JAMA Intern Med. 2013;173(3):218–219. http://dx.doi.org/10.1001/jamainternmed.2013.1229, PMid:23277229 10.1001/jamainternmed.2013.1229

[14] Madhan B, Rajpurohit A, Gayathri H. Attitudes of postgraduate orthodontic students in India towards patient-centered care. J Dent Educ. 2011;75(1):107–114. PMid:21205735

[15] Williams S, Weinman J, Dale J, et al. Patient expectations: What do primary care patients want from the GP and how far does meeting expectations affect patient satisfaction? Fam Pract. 1995;12(2):193–201. http://dx.doi.org/10.1093/fampra/12.2.193, PMid:7589944 10.1093/fampra/12.2.193

[16] Henbest RJ, Fehrsen GS Patient-centredness: is it applicable outside the West? Its measurement and effect on outcomes. Fam Pract. 1992;9(3):311–317. http://dx.doi.org/10.1093/fampra/9.3.311 1459388 10.1093/fampra/9.3.311

[17] Al-Bawardy R, Blatt B, Al-Shohaib S, et al. Cross-cultural comparison of the patient-centeredness of the hidden curriculum between a Saudi Arabian and 9 US medical schools. Med Educ Online. 2009;18:14(19). 10.3885/meo.2009.T0000144 PMC2810096 20101280

[18] Fraser H Doing narrative research: analysing personal stories line by line. Qualitative Social Work. 2004;3(2):179–201. http://dx.doi.org/10.1177/1473325004043383

[19] Markus HR, Kitayama S Culture and the self: implications for cognition, emotion, and motivation. Psychological Review. 1991;98(2):224–253. http://dx.doi.org/10.1037/0033-295X.98.2.224

[20] Horton R African traditional thought and Western science. Journal of the International African Institute. 1967;37(1):50–71. http://dx.doi.org/10.2307/1157195

[21] Omonzejele PF. African concepts of health, disease, and treatment: an ethical inquiry. Explore. 2008;4(2):120–126. http://dx.doi.org/10.1016/j.explore.2007.12.001, PMid:18316055 10.1016/j.explore.2007.12.001

[22] Last M. Another geography: risks to health as perceived in a deep rural environment in Hausaland. Anthropol Med. 2011;18(2):217–229. http://dx.doi.org/10.1080/13648470.2011.591198, PMid:21810038 10.1080/13648470.2011.591198

[23] Knox JR. Exploring the potential for a culturally relevant HIV intervention project: a Swaziland example. Anthropol Med. 2010;17(1):87–98. http://dx.doi.org/10.1080/13648471003607615, PMid:20419519 10.1080/13648471003607615

[24] Mzimkulu KG, Simbayi LC Perspectives and practices of Xhosa-speaking African traditional healers when managing psychosis. Int J Disabil Dev Educ. 2006;53(4):417–431. http://dx.doi.org/10.1080/10349120601008563

[25] Tabuti JR, Dhillion SS, Lye KA Traditional medicine in Bulamogi county, Uganda: its practitioners, users and viability. J Ethnopharmacol. 2003;85(1):119–129. http://dx.doi.org/10.1016/S0378-8741(02)00378-1 12576210 10.1016/s0378-8741(02)00378-1

[26] Janzen JM Ngoma: discourses of healing in central and southern Africa. Berkeley: University of California Press; 1992 http://dx.doi.org/10.1525/california/9780520072657.001.0001

[27] Amanze J The concept of God in Tswana traditional religion In: Amanze J African traditional religions and culture in Botswana. Gaborone, Botswana: Pula Press, 2002; p. 28–53.

[28] Amanze J Witchcraft beliefs and practices In: Amanze J African traditional religions and culture in Botswana. Gaborone, Botswana: Pula Press, 2002; p. 233–245.

[29] Ngoma MC, Prince M, Mann A. Common mental disorders among those attending primary health clinics and traditional healers in urban Tanzania. Br J Psychiatry. 2003;183:349–355. http://dx.doi.org/10.1192/bjp.183.4.349, PMid:14519614 10.1192/bjp.183.4.349

[30] Mercer SW, Reilly D A qualitative study of patient’s views on the consultation at the Glasgow Homoeopathic Hospital, an NHS integrative complementary and orthodox medical care unit. Patient Educ Couns. 2004;53(1):13–18. 15062899 10.1016/S0738-3991(03)00242-8

[31] Yee SK, Chu SS, Xu YM, et al. Regulatory control of Chinese proprietary medicines in Singapore. Health Policy. 2005;71(2):133–149. http://dx.doi.org/10.1016/j.healthpol.2003.09.013, PMid:15607377 10.1016/j.healthpol.2003.09.013

[32] Xu J, Yang Y. Traditional Chinese medicine in the Chinese health care system. Health Policy. 2009;90(2–3):133–139. http://dx.doi.org/10.1016/j.healthpol.2008.09.003, PMid:18947898 10.1016/j.healthpol.2008.09.003 PMC7114631

[33] Frankel RM, Devers KJ. Study design in qualitative research--1: developing questions and assessing research needs. Educ Health. 2000;13(2):251–261. http://dx.doi.org/10.1080/13576280050074534, PMid:14742087 10.1080/13576280050074534

